# Structure–Antioxidant Activity Relationship of Polysaccharides Isolated by Microwave/Ultrasonic-Assisted Extraction from *Pleurotus ferulae*

**DOI:** 10.3390/antiox14010091

**Published:** 2025-01-14

**Authors:** Hongjin Zhou, Zhongxiong Fan, Yuan Li, Xuelian Liu, Bo Wang, Jianguo Xing, Jiang He, Ruifang Zheng, Jinyao Li

**Affiliations:** 1 Xinjiang Key Laboratory of Biological Resources and Genetic Engineering, College of Life Science and Technology, Xinjiang University, Urumqi 830017, China; hjzhou@stu.xju.edu.cn (H.Z.); wbxjhyj@xjmu.edu.cn (B.W.); 2 School of Pharmaceutical Sciences, Institute of Materia Medica, Xinjiang University, Urumqi 830017, China; fanzhongxiong@xju.edu.cn (Z.F.); liyuanstc@xju.edu.cn (Y.L.); lxl_nuo@xju.edu.cn (X.L.); 3 Xinjiang Key Laboratory of Uygur Medical Research, Xinjiang Institute of Materia Medica, Urumqi 830004, China; xjguodd@163.com (J.X.); hj_1211@163.com (J.H.)

**Keywords:** structure–activity relationship, *Pleurotus ferulae*, polysaccharides, antioxidant activity

## Abstract

To investigate the structure–antioxidant activity relationship, *Pleurotus ferulae* polysaccharides were extracted using ultrasonic (U-PFPS) and microwave/ultrasonic-assisted methods (MU-PFPS). Compared to U-PFPS with a molecular weight of 1.566 × 10^3^ kDa, MU-PFPS exhibited a lower molecular weight of 89.26 kDa. In addition, unlike U-PFPS, which is primarily composed of glucose (Glu:Man:Gal = 91.1:3.5:5.4), MU-PFPS has a more balanced composition of Glu:Man:Gal in the ratio of 39.4:27.8:32.8 and contains more branched chains. Furthermore, antioxidant analysis revealed that high concentration (at concentrations above 600 μg/mL) MU-PFPS demonstrated stronger protective effects against oxidative damage in RAW264.7 cells than U-PFPS did. Collectively, these data suggest that lower molecular weight and higher branching degree of polysaccharides at appropriate concentrations may correlate with enhanced antioxidant enzyme activities. Our work provides a method for isolating polysaccharides with higher antioxidant activity and offers insights into the structure–activity relationship of polysaccharides, laying the foundation for future applications in polysaccharide modification and structural characterization.

## 1. Introduction

Polysaccharides obtained from the same resource using different extraction methods exhibit diverse structural characteristics and biological activities [1]. For instance, *Cordyceps sinensis* polysaccharides with molecular masses ranging from 3000 to 10,000 kDa exhibit higher antitumor activity [2]. Extracted from rice bran using hot water, polysaccharides with varying molecular masses display different antioxidant properties [3]. Similarly, *Zingiber officinale* polysaccharides extracted by hot water, ultrasonic-assisted, and enzyme-assisted methods have similar monosaccharide compositions but differ in glycosidic linkage types, resulting in distinct anti-fatigue, antioxidant, and antitumor activities [4,5,6]. Studies on the structure–activity relationship (SAR) of polysaccharides indicate that different preliminary characteristics, such as molecular mass, degree of branching, and degree of polymerization, greatly influence their conformation and polydispersity index [7,8].

*Pleurotus ferulae*, an edible and medicinal mushroom predominantly found in Xinjiang, China, has been studied for its immunomodulatory properties for a long time [9]. Our previous research showed that ultrasonic-assisted water extracts of *P. ferulae* promoted dendritic cell (DC) maturation, primarily due to its polysaccharide content [10]. Furthermore, purified *P. ferulae* polysaccharides (PFPS) stimulated DC and macrophage activation via the Toll-like receptor 4 (TLR4) signaling pathway, activated T helper cell (Th1) responses, and enhanced antigen-specific cellular immune responses induced by DC-based vaccines [11]. These findings demonstrate that polysaccharides not only enhance innate and adaptive immunity but also improve vaccine-induced antigen-specific immune responses as adjuvants. Recent studies, including our own, have reported various biological activities of polysaccharides isolated from natural products, such as antitumor [12,13], antioxidant [14,15], and hypoglycemic activities [16,17]. The biological activities of polysaccharides are related to their physicochemical characteristics [18], bioavailability [19], and conjugation sites [20]. Therefore, research has focused on the SAR of polysaccharides, as well as their basic structure and advanced conformation, have becomes the key issues of the study.

One group reported that PFPS extracted by ultrasonic-assisted methods exhibited a flexible single-stranded conformation at low concentrations and more polymers at higher concentrations [21]. Another study showed that crude polysaccharides from *P. ferulae* obtained by hot water extraction had stronger antioxidant activity than purified fractions [22], indicating that *P. ferulae* contains polysaccharides with potent antioxidant activity. To further explore the SAR of PFPS, different extraction methods are needed to isolate PFPS.

Given that microwave extraction significantly affects the structural characteristics and exhibits remarkable antioxidant activities of several types of polysaccharides [23,24,25], ultrasonic or microwave/ultrasonic-assisted extractions were employed to isolate polysaccharides from *P. ferulae*, yielding U-PFPS and MU-PFPS, respectively. The molecular weight, monosaccharide composition, branching degree, and antioxidant activity of U-PFPS and MU-PFPS were compared to explore the SAR of PFPS. This study also provides a new method for isolating PFPS with strong antioxidant activity, contributing to the application and utilization of PFPS and related polysaccharides.

## 2. Materials and Methods

### 2.1. Materials and Reagents

1,1-diphenyl-2-picryhydrazyl (DPPH) was purchased from Shanghai Lanji Technology Co., Ltd. (Shanghai, China). A glutathione peroxidase (GSH-Px) kit and superoxidase dismutase (SOD) kit were purchased from Beyotime (Beyotime, Shanghai, China). A catalase (CAT) kit was obtained from Nanjing Jiancheng Bioengineering Institute (Nanjing, China). RAW 264.7 cells were obtained from ATCC (Manassas, VA, USA). DMEM medium was purchased from Gibco (ThermoFisher, Waltham, MA, USA). Hydrogen peroxide (H_2_O_2_) and phenol were obtained from Xinjiang Key laboratory of Biological Resources and Genetic Engineering. Penicillin–streptomycin and fetal bovine serum were purchased from Thermo Fisher Scientific (Waltham, MA, USA). All other chemicals and reagents were of analytical grade unless otherwise specified.

### 2.2. Preparation of Polysaccharides

#### 2.2.1. Extraction and Purification of U-PFPS and MU-PFPS

The method of extracting crude polysaccharides is shown in Supplementary Figures S1 and S2. *Pleurotus ferulae* were purchased from Nanshan (Urumqi, Xinjiang) and identified by professor Jiang He from the Xinjiang Institute of Materia Medica (Key Laboratory of Xinjiang Uygur Medicine, Urumqi, China). According to our previous study [9,11], crude polysaccharides were collected through ultrasonic-assisted method with some modifications. Microwave/ultrasonic-assisted polysaccharide was extracted with additional microwave treatment at different power. The effects of power (210, 280, 350, 560, and 700 W) and duration (0, 2, 5, 8, and 10 min) on polysaccharide content were studied by single factor experiment at the ratio of solid to liquid of 1:10. The power and microwave duration were selected at that corresponding with highest yield of polysaccharides to apply to following purification. The yield was calculated according to the formula:

(1) $$Yield\left(\%\right)=\left(\frac{W}{W_{0}}\right)\times 100$$

where *W*_0_ is the weight of *Pleurotus ferulae* dried powder and *W* is the weight of polysaccharide extraction after freeze drying.

The U-PFPS and MU-PFPS were purified from two kinds of crude polysaccharides through DEAE-52 cellulose and eluted with 0 M, 0.1 M, 0.2 M, and 0.5 M NaCl solution with modification [9]. Plot elution curves of polysaccharide absorbance at 490 nm were used for quantification as described below, with number of tubes used instead of retention time.

#### 2.2.2. Quantification of Polysaccharides and Protein

Polysaccharide content was detected by phenol-sulfuric acid method [26]. A glucose solution with different concentration (0, 2, 5, 10, 20, 25, 50, and 100 μg/mL) was used as standard solution; 100 μL of aqueous solution was mixed with 200 μL of 5% phenol (*w*/*v*) and 500 μL of sulfuric acid. The solutions were maintained at 37 °C for 30 min, then placed at room temperature for 5 min, and absorption was measured at a 490 nm wavelength. Furthermore, polysaccharide content was calculated according to the formula:

(2) $$Polysaccharide\;content\left(\%\right)=\left(\frac{C\cdot V}{C_{0}\cdot V_{0}}\right)\times 100$$

where *C* is the polysaccharide concentration calculated according to standard curve, C_0_ is the original polysaccharide concentration, 1000 μg/mL, and *V* = *V*_0_ = 100 μL.

To measure the content of protein, 2 mg/mL polysaccharide solution was detected by BCA Protein Assay Kit (ThermoFisher, Waltham, MA, USA) according to the manufacturer’s instruction. BCA ratios of 0, 0.16, 0.40, 0.80, 1.20, 1.60, and 2.00 mg/mL were prepared as standard solutions. Samples of 25 μL were mixed with 200 μL work solution and maintained at 37 °C for 30 min, then cooled to room temperature for 30 min, and absorption was measured at a 562 nm wavelength. The protein content was calculated according to the formula:

(3) $$Protein\;content\left(\%\right)=\left(\frac{C_{1}\cdot V_{1}}{C\cdot V}\right)\times 100\%$$

where *C* is the protein concentration calculated according to standard curve, *C*_1_ is the protein concentration of polysaccharide sample, and *V* = *V*_1_ is the volume of the sample.

### 2.3. Structure Analysis

#### 2.3.1. Determination of Molecular Weight of U-PFPS and MU-PFPS

Molecular weights of MU-PFPS and U-PFPS were examined using high-performance gel permeation chromatography (HPGPC) detected by Alliance HPLC (LC-20, Shimadzu Corporation, Kyoto, Japan). The TSK-GEL G3000 PW_XL_ column (TOSHO, Kyoto, Japan 7.8 mm × 300 mm, id.) and RI detector (RID-20A, Shimadzu Corporation, Kyoto, Japan) were maintained at 40 °C. Dextran (SIGMA-ALDRICH, St. Louis, MO, USA) with different molecular weights (5, 50, 70, 80, 150, and 500 kDa) was used to plot the standard curve based on the retention time and logarithm of relative molecular mass. Samples were prepared at a concentration of 1.0 mg/mL through a 0.22 μm water filter. The molecular weights of MU-PFPS and U-PFPS were calculated by a calibration curve equation according to retention time.

MU-PFPS-1 separated through Sephadex LH20 was examined using high-performance liquid chromatography (HPLC) detected by Alliance HPLC (Waters e2695, Waters, Milford, MA, USA). The Waters Ultrahydrogel column (Waters, USA) and RI detector were maintained at 40 °C. Dextran with different molecular weights (5.2, 11.6, 18.3, 48.6, 148, 273, 410, and 668 kDa) were used to plot the standard curve based on the retention time and logarithm of relative molecular mass. Samples were prepared at a concentration of 1.0 mg/mL through a 0.22 μm water filter. The molecular weights were calculated by a calibration curve equation according to retention time.

#### 2.3.2. Monosaccharide Composition Analysis of U-PFPS and MU-PFPS

The monosaccharide compositions were determined by aldononitrile acetate method and analyzed by GC as in a previous study [27] with modification. In brief, the sample (4 mg) was hydrolyzed with 2 M TFA at 110 °C for 4 h. After TFA was removed by methanol through rotary evaporation, the hydrolysate was reduced by 8 mg hydroxylamine hydrochloride and acetylated with 0.5 mL acetic anhydride at 90 °C for 30 min. Similarly, D-mannose, D-glucose, and D-galactose were used as monosaccharide standards through aldononitrile acetate derivatization. The final derivative was dissolved in dichloromethane and analyzed by SHIMADZU GC-2014C (SHIMADZU, Kyoto, Japan) on a Wondacap column. The column temperature was set at 160 °C for 5 min, then raised to 230 °C at 5 °C/min. The injector and detector were fixed at 250 °C. The compositions of monosaccharide were qualitatively and semi-quantitatively determined along with retention time according to the standard monosaccharide curve.

#### 2.3.3. Atomic Force Microscopy

The MU-PFPS and U-PFPS were respectively dissolved and diluted with distilled water as 10 μg/mL. After passing through 0.22 μm membranes, 5 μL of each sample was dropped on the surface of mica sheet and dried at room temperature. The molecular morphology of polysaccharides was observed by using atomic force microscopy (Bruker Dimension icon, Bruker, Germany). Nanoscope software (Nanoscope v1.40r1, Bruker, Billerica, MA, USA) was used to analyze AFM images. The surface roughness and other parameters (Z range, Ra, skewness, and kurtosis) of polysaccharide in a dry state were measured.

#### 2.3.4. Fourier Transform Infrared Spectra (FT-IR)

Masses of 2 mg of U-PFPS and MU-PFPS were respectively mixed with 400 mg of KBr, ground, and pressed into tablets. Scans were conducted from 4000 cm^−1^ to 400 cm^−1^ at a resolution of 2.0 cm^−1^. FT-IR spectra of U-PFPS and MU-PFPS were recorded on a Vertex 70 RAM II spectrophotometer (Bruker, Billerica, MA, USA).

### 2.4. Determination of Antioxidation Activity

#### 2.4.1. Assay of DPPH Scavenging Activity

The measurement of DPPH· scavenging activity was gauged using the method described by Lee et al. [28]. The DPPH· solution was prepared by dissolving 6 mg of the compound in 100 mL ethanol. Samples were diluted with distilled water as 1 mg/mL and mixed with isometric DPPH· solution. With distilled water and isometric ethanol as blank, the absorbance was determined by spectrophotometer at a 517 nm wavelength after reaction. The free radical scavenging activity was calculated as follows:

(4) $$DPPH\cdot radical\;scavenging\;activity\left(\%\right)=\left(1-\frac{A_{2}-A_{1}}{A_{0}}\right)\times 100$$

where *A*_0_ is the absorbance of the blank group, *A*_1_ is the absorbance of isometric ethanol and DPPH· solution as standard group, and *A*_2_ is the absorbance of the sample group. A volume of 100 μg/mL vitamin C (Vc) was used as positive control.

#### 2.4.2. Assay of ABTS· Scavenging Activity

According to a published method [29], the ABTS· radical was prepared by reacting an ABTS solution (7.0 mM) with a potassium persulfate aqueous solution (2.45 mM) at 4 °C in darkness for 12 h. The ABTS· solution was diluted with distilled water (5 mM) and equilibrated at 37 °C for 30 min. Samples were separately added to 2.0 mL of the diluted ABTS· solution. The mixture was allowed to react at room temperature for 10 min, and the absorbance was measured at 734 nm. The scavenging activity of the ABTS radical was obtained by the following equation:

(5) $$\mathrm{ABTS}\cdot \;\mathrm{radical}\;\mathrm{scavenging}\;\mathrm{activity}\;\left(\%\right)=(1-\frac{A_{1}{-A}_{2}}{A_{0}})\times 100$$

where *A*_0_ is the absorbance of the blank group, *A*_1_ is the absorbance of the sample group, and *A*_2_ is the absorbance of the mixed solution without ABTS·. Each experiment was conducted in triplicate.

#### 2.4.3. Assay of Superoxide Anion Free Radical (O_2_^·^) Scavenging Activity

Referring to a published method [30], the scavenging activities of polysaccharides were measured using the following method with slightly modification. The polysaccharide solution (1 mg/mL) was diluted with deionized water and Tris-HCl buffer solution (2.5 mL, 0.1 mol/L, pH = 8.2) to 100 μg/mL, and the mixture maintained for 20 min at 25 °C. The pyrogallol solution (50 μL, 0.05 mol/L), maintained at 25 °C as well, was added to initiate the autoxidation reaction. The rate was reflected by the absorbance at 319 nm, which was recorded at an interval of 30 s for 5 min on a spectrophotometer. The capability of scavenging O_2_^·^ was calculated as follows:

(6) $$O_{2}^{\cdot -}\;\mathrm{radical}\;\mathrm{scavenging}\;\mathrm{activity}\;\left(\%\right)=\frac{A_{0}-A_{1}}{A_{0}}\times 100$$

where the absorbance value of the polysaccharide sample group is *A*_1_, while the absorbance value of the blank control group is *A*_0_.

#### 2.4.4. Method of Cytotoxicity Experimentation

The possible toxic effects of U-PFPS and MU-PFPS treatment on the proliferation of macrophages were measured using the MTT (3-(4,5-Dimethylthiazol-2-yl)-2, 5-diphenyltetrazolium bromide) method as described earlier by Generalov et al. [31]. RAW 264.7 cells were obtained from ATCC (USA). The cells were grown in DMEM medium containing 10% fetal calf serum and 100 μg/mL penicillin and streptomycin. The cells were seeded into 96-well plates at a density of 2 × 10^5^ cells/well, and different concentrations of the medium of MU-PFPS and U-PFPS were added (100, 200, 400, 600, 800, and 1000 μg/mL), and cultured at 37 °C with 5% CO_2_ for 24 h. The cell viability was analyzed with MTT method and the absorbance at 450 nm was measured on a BioTek Epoch Microplate Spectrophotometer (Agilent, Santa Clara, CA, USA).

#### 2.4.5. Viability Assay for H_2_O_2_-Induced Oxidative Damage RAW 264.7 Cell Model

Cell viability was determined by a MTT assay according to the treatments described above. RAW 264.7 cells were seeded into 96-well plates at a density of 2 × 10^5^ cells/well and cultivated for 24 h. For screening the appropriate H_2_O_2_ concentration, the serum-free DMEM medium with different concentration of H_2_O_2_ (100, 200, 400, 600, 800, 1000, and 1200 μg/mL) were added as injury model groups, and same cell medium without H_2_O_2_ was taken as control group. After cultivation for 8 h, the MTT reagent was added to each well and measured at an absorbance of 490 nm on the BioTek Epoch Microplate Spectrophotometer (Agilent, Santa Clara, CA, USA).

#### 2.4.6. Protective Effects of Polysaccharides on H_2_O_2_-Induced Oxidative Damage in RAW264.7 Cells

A protective effect assay of polysaccharides was performed according to a reported method [32]. H_2_O_2_-induced oxidative damage RAW264.7 cells were pretreated with 100 μg/mL Vc and different concentrations of MU-PFPS and U-PFPS, respectively, for 12 h in new serum-free medium; then, 544 μM H_2_O_2_ was added to each group except the control group and cultivated for 8 h. After that, the 10 μL CCK-8 reagent was added and the absorbance was measured at 450 nm on the BioTek Epoch Microplate Spectrophotometer (Agilent, Santa Clara, CA, USA).

According to protective effect of different eluent fluids of polysaccharides extracted by different methods, the different concentrations of polysaccharides were added to treated groups and the protective effects were measured at an absorbance of 450 nm by BioTek Epoch Microplate Spectrophotometer (Agilent, Santa Clara, CA, USA) as well.

#### 2.4.7. Determination of SOD, CAT, GSH-Px and MDA

The oxidative activity in the cells was determined according to previously reported methods with slight modification [32,33]. RAW 264.7 cells were seeded into 6-well plates at a density of 1 × 10^6^ cells/well and cultivated for 24 h. Similarly, the cells were pretreated with 100 μg/mL Vc and different concentrations of MU-PFPS and U-PFPS, respectively, for 12 h in new serum-free medium, then treated with 544 μM H_2_O_2_ for 8 h. The supernatant and precipitate were separately collected by centrifuge at 1200 rpm for 7 min. The levels of MDA, SOD, CAT, and GSH-Px were determined using commercially available kits (Beyotime, Shanghai, China).

#### 2.4.8. Determination of ROS by Flow Cytometry

According to a reported study [34], the ROS level in the cells was determined as following process. RAW 264.7 cells were seeded into 24-well plate and cultivated for 24 h. After being exposed to 100 μg/mL Vc and different concentration of MU-PFPS and U-PFPS (100, 600, and 1000 μg/mL), the cells were exposed to 544 μM H_2_O_2_ for 8 h. Disposed supernatant and collected cells were centrifuged at 1200 rpm for 7 min, then the cells were resuspended by PBS and recentrifuged. The serum-free DMEM medium with DCFH-DA 2, 7-dichlorofuorescin diacetate (DCFH-DA) at a ratio of 5000:1 were blended with cells and incubated for 30 min at 37 °C, while reversely oscillated every 5 min. Subsequently, the process was terminated with PBS and cells collected by centrifuging. The proportion of positive cells was detected by a CytoFLEX fluorescence spectrophotometer (Beckman, Indianapolis, IN, USA).

### 2.5. Quantitative Real-Time PCR

RAW264.7 cells were seeded in 6-well plates. The total RNA of each treatment group was isolated through MolPure cell RNA kit (Yeasen, Shanghai, China) according to the manufacturer instructions. Total RNA was reverse transcribed to complementary DNA according to the manufacturer instructions (EasyScript Onestep gDNA removal and cDNA Synthesis supermix, TransGen, Beijing, China). PCR was conducted on an ABI 7300 Fast Real-time PCR system (QuantStudio 3, ThermoFisher Scientific, Waltham, MA, USA) using Real-time PCR Easy SYBRGREEN1 (Foregene Co., Ltd., Sichuan, China). Three replicates were performed per sample, and the relative mRNA expression levels of *Nrf2* and *HO-1* genes were determined using the comparative Ct method (2^−ΔΔCt^), normalized to GADPH expression.

Primers and sequences

**Primer Name**

**Sequence**
*Nrf2*-FCCAGCACAACACATACCA*Nrf2*-RTAGCCGAAGAAACCTCATT*HO-1*-FGAACGCAACAAGGAGAAC*HO-1*-RCTGGAGTCGCTGAACATAG

### 2.6. Statistical Analysis

All experiments were performed in triplicate and results expressed as mean ± standard deviation (SD). The results of flow cytometry were analyzed by FlowJo (Tree Star, Inc., Ashland, OR, USA) and the statistical analysis was performed by a one-way ANOVA test using GraphPad Prim 8.0 (Graph-Pad Software, San Diego, CA, USA). Values of *p* < 0.05 were evaluated as statistically significant. Standard deviation and statistical significance were calculated using Prism 8.0 (GraphPad, San Diego, CA, USA).

## 3. Results and Discussion

### 3.1. Extraction, Purification and In Vitro Antiradical Activity Assay of Polysaccharides

#### 3.1.1. Yield and DPPH· Radical Scavenging Activities of Crude Polysaccharides

**Figure 1:**
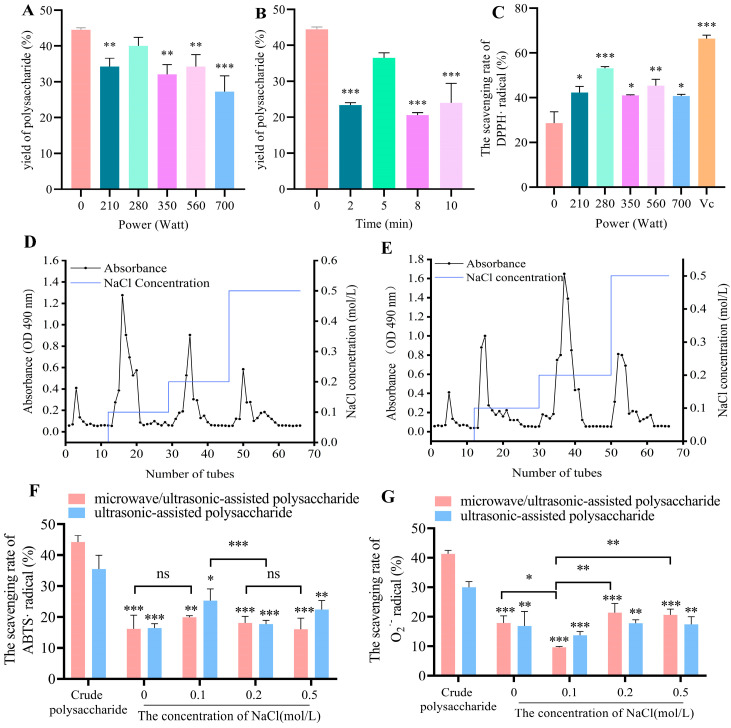
Effect of (**A**) different microwave power and (**B**) microwave duration on the extraction yield of MU-PFPS. (**C**) Scavenging rate of DPPH radical. * *p* < 0.05, ** *p* < 0.01, *** *p* < 0.001 vs. control. (**D**) Elution curves of microwave/ultrasonic-assisted polysaccharides and (**E**) ultrasonic-assisted polysaccharides. In vitro antioxidant assays: (**F**) ABTS· scavenging ability; (**G**) O_2_^·−^ scavenging ability. * *p* < 0.05, ** *p* < 0.01, *** *p* < 0.001, ns = no significance.

Due to enhanced mass transfer and cell disruption, ultrasonic-assisted extraction (UAE) has been widely used despite its inhomogeneous heating process. According to our previous reports [9,10], the yield of crude U-PFPS extracted by UAE at 700 W reached 44%. To accelerate the extraction process, microwave was combined with UAE [24,35]. The yields of microwave/ultrasonic-assisted crude polysaccharides (crude MU-PFPS) extracted at microwave powers of 210 W, 280 W, 350 W, 560 W, and 700 W are shown in Figure 1A. Surprisingly, microwave/ultrasonic synergistic extraction resulted in lower polysaccharide yields than UAE, with the maximum yield being 40% at 280 W. Additionally, the extraction yield gradually increased within 2 to 5 min and peaked at 5 min before decreasing (Figure 1B). Although microwave-assisted extraction (MAE) reduces time consumption, it typically operates at high temperatures under alternating electromagnetic fields, causing polar substances to undergo strong polarity oscillations, leading to structural bond breakdown and hydrogen bond relaxation, thereby damaging glycosidic linkages as microwave power and time increase [36]. Moreover, the leaching and diffusion of shorter polysaccharide chains reduce yield during purification. Thus, the optimal condition for MU-PFPS with high yield was also 280 W for 5 min.

DPPH radicals are extensively used to evaluate natural antioxidants [37]. The effect of microwave power on the in vitro antioxidant activity of crude MU-PFPS was investigated based on DPPH scavenging capacity. As shown in Figure 1C, DPPH scavenging capacity increased with microwave power from 210 W to 280 W. After treatment at powers exceeding 280 W for 5 min, the DPPH scavenging rate decreased. This trend suggests that the antioxidant activity of crude MU-PFPS significantly increased at 280 W for 5 min but decreased with continuous fractionation. The DPPH scavenging ability correlated with the content of crude polysaccharides. Therefore, the optimal condition for crude MU-PFPS extraction was 280 W for 5 min.

#### 3.1.2. Effects of In Vitro Antiradical Activity of Purified Polysaccharides

To further evaluate potential antioxidant properties and investigate the relationship between antioxidant activity and structural characteristics, polysaccharides were purified from crude U-PFPS and MU-PFPS using DEAE-52 cellulose and eluted with 0 M, 0.1 M, 0.2 M, and 0.5 M NaCl solutions (Figure 1D,E). Fractions were collected and measured for polysaccharide contents using the phenol-sulfate method (Table 1). According to the elution curve, the two crude polysaccharides extracted by different methods showed distinct elution characteristics. The peak fractions of MU-PFPS were obtained at 0.2 M NaCl, while those of U-PFPS were obtained at 0.1 M NaCl. The elution curve of U-PFPS aligns with our previous study [9,10]. Purification by DEAE-52 is based on binding to positively charged ions on the resin, acting as a molecular sieve. Therefore, the elution peaks correspond to polysaccharides with different ion potentials and molecular weights. Consequently, the antioxidant activities of these fractions were compared.

As shown in Figure S3, the concentration-dependent behavior of DPPH· radical scavenging was measured at 1000 μg/mL. Crude MU-PFPS exhibited higher scavenging rates than its fractions, while there was no significant difference in DPPH· free radical scavenging rates among U-PFPS fractions eluted with different NaCl solutions. Since U-PFPS is a water-soluble polysaccharide that hardly dissolves completely in ethanol, measuring its antioxidant activity using DPPH· scavenging rate in organic solvents is inadequate. Instead, the ABTS· decolorization assay in aqueous medium is more suitable for assessing the antioxidant capacity of polysaccharide samples. As depicted in Figure 1F, 0.1 M eluted U-PFPS had better ABTS· scavenging ability compared to other elution phases, but there was no significant difference among MU-PFPS elution phases. To further measure the antioxidant activity of MU-PFPS and U-PFPS, the scavenging rate of O_2_·, one of the precursors of highly reactive oxygen species (ROS), was also detected (Figure 1G). Compared with the scavenging ability of DPPH, the overall scavenging of O_2_· was relatively weaker. Due to the presence of electrophilic groups, it is easier for hydrogen release from O-H bonds than from stabilized O_2_· [38]. The O_2_· scavenging rates of crude MU-PFPS and U-PFPS were higher than those of their respective fractions. The order of O_2_· scavenging rates for MU-PFPS and U-PFPS eluted with different NaCl solutions was 0.2 M > 0.5 M > 0 M > 0.1 M. Notably, the lowest O_2_· scavenging rate of MU-PFPS eluted at 0.1 M NaCl solution might be related to its specific molecular structure. Considering both yield and polysaccharide contents (as shown in Table 1), MU-PFPS eluted at 0.2 M NaCl solution and U-PFPS at 0.1 M NaCl solution were chosen for further structural investigation and antioxidant effect studies in cells.

Indeed, according to Table 1, small molecules other than polysaccharides and proteins (such as phenols, aldehydes/ketones, or terpenes), although they may account for a relatively small proportion, could be key contributors to the better free radical scavenging effect of crude polysaccharides compared to purified polysaccharides. However, during polysaccharide extraction, most of these substances are eliminated by alcohol precipitation, thereby highlighting the intrinsic free radical scavenging ability of the polysaccharides themselves. As previously reported [39], the potential of polysaccharides for radical scavenging is influenced by molecular weight, monosaccharide composition, and higher-order structure. Unlike other polysaccharides, MU-PFPS and U-PFPS did not exhibit concentration-dependent behavior in radical scavenging. This may be linked to the presence of keto or aldehyde groups that influence hydrogen-donating ability [40]. Furthermore, molecular weight can significantly enhance radical scavenging potential due to helical structure integrity or surface area [41]. We wondered what structural characteristics lead to the different antiradical activities observed between the two polysaccharides. Subsequent assays revealed differences between MU-PFPS and U-PFPS.

### 3.2. Preliminary Structure Characterization of MU-PFPS and U-PFPS

#### 3.2.1. Monosaccharide Composition Assay

**Figure 2:**
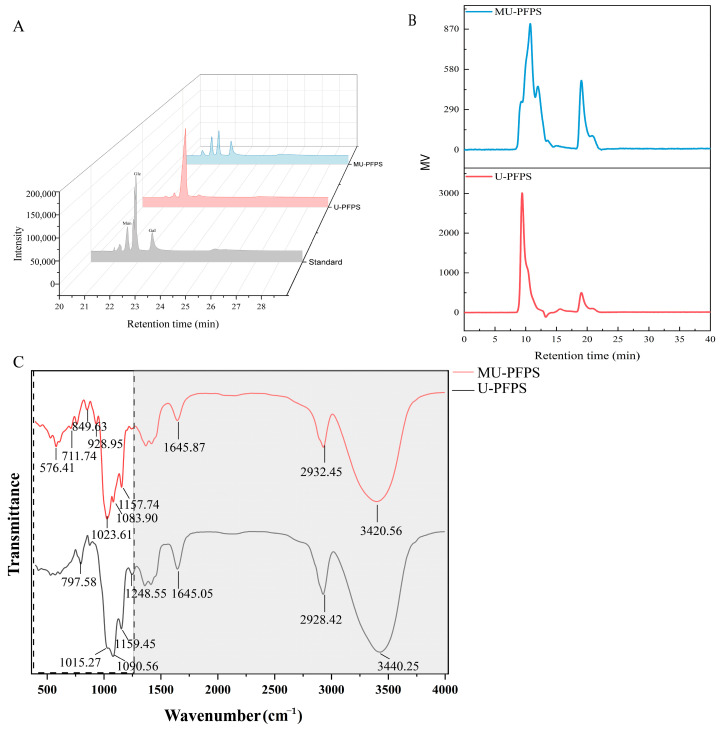
(**A**) GC chromatograms of standard monosaccharides, U-PFPS and MU-PFPS. Relative molar ratio was calculated by area normalization. (**B**) HPGPC chromatograms of U-PFPS and MU-PFPS. (**C**) FT-IR of U-PFPS and MU-PFPS. The shaded areas represent the positions where there is no significant difference in peak emergence.

The monosaccharide compositions of MU-PFPS and U-PFPS were determined by GC chromatography after PMP derivatization to address unusual resistance to acid hydrolysis, decarboxylation, and lactonization when uronic acid is liberated from the polymer [27]. As shown in Figure 2A, monosaccharides were successfully separated within 25 min, and the peaks were identified as mannose, glucose, and galactose by comparing retention times with standard monosaccharides. Both MU-PFPS and U-PFPS were primarily composed of D-mannose, D-glucose, and D-galactose, albeit in different ratios (Table 2). U-PFPS was mainly composed of glucose (91.1%), followed by mannose (3.5%) and galactose (5.4%). In contrast, MU-PFPS had similar proportions of glucose (39.4%), mannose (27.8%), and galactose (32.8%). Interestingly, microwave-assisted extraction did not improve the extraction yield of PFPS but generated volumetrically distributed fractions, suggesting that microwaves primarily convert energy into thermal effects [36]. Thus, different extraction methods may not alter the monosaccharide composition but rather their relative contents instead [42]. These results indicate that U-PFPS might have a backbone analogous to glucan with specific intrachain monosaccharide components, whereas MU-PFPS mainly consisted of discrepant monomer compositions. Generally, this suggests that U-PFPS has a lower degree of branching and higher molecular weight, whereas MU-PFPS has more glycosidic linkages, terminal, and branch types. These physical characteristics contribute to scavenging or antioxidant activities, respectively. To further verify these structural conjectures, molecular weight and 3D structure measurements were conducted.

#### 3.2.2. Molecular Weight Measurement

The average molecular weights (Mw) of U-PFPS and MU-PFPS were measured using HPGPC (Figure 2B). Retention times and molecular weight values are presented in Table 3. The HPGPC profile of U-PFPS showed a single peak, indicating homogeneity, consistent with our previous study [38]. However, the profile of MU-PFPS showed two peaks. We attempted to separate MU-PFPS using Sephadex LH20, based on a standard curve derived from retention time and logarithm of relative molecular mass. The standard equation was y = 0.1207X^2^ − 3.4206X + 27.968 (R^2^ = 0.9997, y = lg M_w_, x = R_t_). The results (Table 4) indicated that peak 1 was the main component (mass ratio > 80%). However, secondary purification significantly reduced yield (<30%) while maintaining high purity (Figure S4). These two peaks were difficult to separate, so they were collected together to obtain high yields of MU-PFPS. Based on the standard curve from retention times of different standard dextrans, which equation was as y = 0.1235X^2^ − 3.4338X + 27.578 (R^2^ = 0.9927, y = lg M_w_, x = R_t_), the estimated average molecular weights were 1.566 × 10^3^ kDa for U-PFPS, 89.26 kDa for MU-PFPS-1, and 14.76 kDa for MU-PFPS-2. Combined with different antioxidant activities, these results suggest that lower molecular weight contributes to higher protective effects on oxidative damage cells due to altered physicochemical properties after different extraction methods [43].

#### 3.2.3. FT-IR

By comparing the infrared spectra of MU-PFPS and U-PFPS (as depicted in the Figure 2C), both show a broad and intense absorption peak within the range of 3350–3500 cm^−1^, which is attributed to the -OH stretching vibration peak. Similar situations also arise around 2925–2935 cm^−1^, indicating the stretching vibration peaks of C-H, and 1645 cm^−1^, ascribable to bending of water. Nevertheless, the absorption peak of U-PFPS near 1248 cm^−1^ is stronger than that of MU-PFPS, suggesting the arise from CH2 rocking and indicating that the multiple branches of MU-PFPS are likely to be short chains. Additionally, the strong absorption peaks of U-PFPS at 1090 cm^−1^ and 1015 cm^−1^ further verify the possibility of at least one long chain. In contrast to MU-PFPS, the absorption peaks in this range shift to lower wavenumbers and the stretching vibration weakens, suggesting the formation of intermolecular hydrogen bonds, which further supports the possibility that MU-PFPS has multiple short branches. In the fingerprint region, the absorption peak of U-PFPS at 797 cm^−1^ indicates δ(C-H), while MU-PFPS exhibits characteristic absorption peaks such as γ(C-H), δ(C-O), and γ(C-O). Furthermore, considering that there is no shift or intensity difference in the absorption peaks of U-PFPS and MU-PFPS in the regions indicating carboxyl groups at 3500 cm^−1^, 1650 cm^−1^, and 1300–1430 cm^−1^, it implies that the structural differences between MU-PFPS and U-PFPS may be more concentrated on the number and length of branches, and the different degrees of exposure of terminal glycosidic groups lead to differences in the formation of intramolecular and intermolecular hydrogen bonds.

#### 3.2.4. AFM

**Figure 3:**
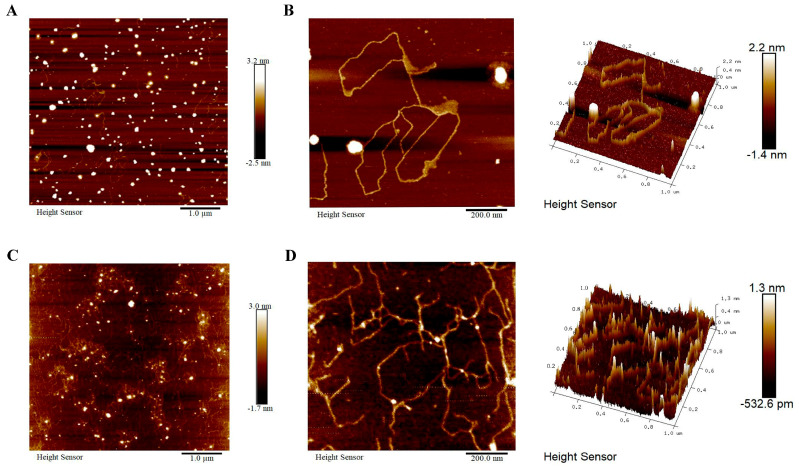
Optical density of U-PFPS (**A**) and MU-PFPS (**C**) at a range of 5.0 μm × 5.0 μm. Planar and 3D AFM images of U-PFPS (**B**) and MU-PFPS (**D**) at a range of 1.0 μm × 1.0 μm. Concentration of polysaccharides was 10 mg/mL.

To investigate the higher-order structure, AFM was used to scan the surface morphology by measuring interaction forces between molecules on the sample surface and microcantilever probe [44]. The planar and three-dimensional structures of U-PFPS and MU-PFPS at 10 μg/mL were analyzed. Planar morphology scans over a 5.0 μm × 5.0 μm area indicated that U-PFPS formed more aggregates (Figure 3A) compared to MU-PFPS (Figure 3C). Micrographs (1.0 μm × 1.0 μm) showed that U-PFPS tended to form single flexible chains with low branching degree (Figure 3B). Planar images clearly showed that MU-PFPS was more homogeneous with fewer aggregates, as indicated by the z range and standard deviation (Table 5). U-PFPS exhibited an asymmetric linear structure with few branches, as evidenced by skewness and kurtosis. Heights of U-PFPS ranged from 0.4 to 2.2 nm, while those of MU-PFPS ranged from 0.4 to 1.3 nm (Figure 3D). Additionally, U-PFPS showed higher roughness than MU-PFPS. These results suggest that U-PFPS tends to form aggregates due to its flexible single-chain structure, while MU-PFPS, with more branches, reduces aggregation.

### 3.3. Protective Effect of U-PFPS and MU-PFPS on H_2_O_2_-Induced Oxidative Damage in RAW264.7 Cells

**Figure 4:**
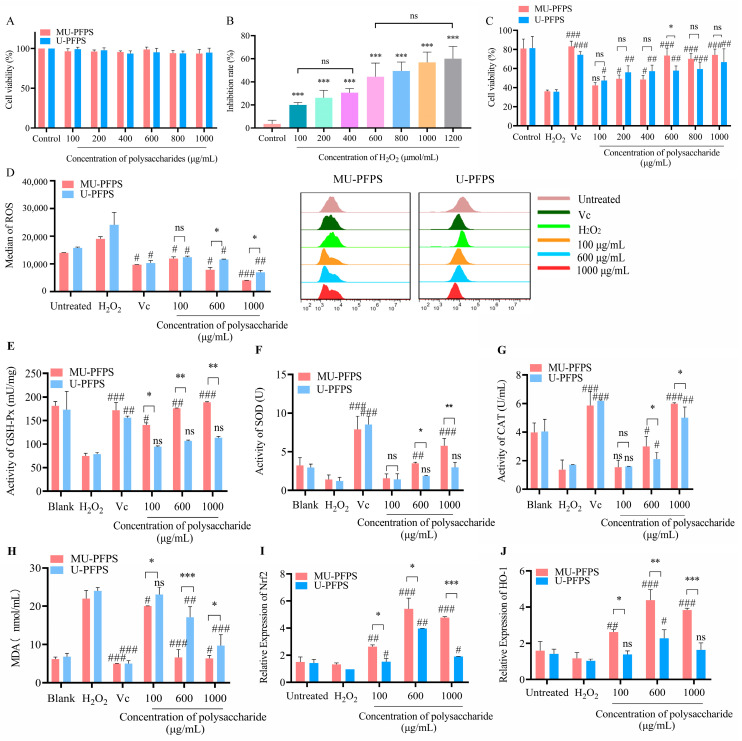
(**A**) Effect of MU-PFPS and U-PFPS on cell viability of RAW264.7 cells by MTT assay. (**B**) Effect of H_2_O_2_ with different concentration on RAW264.7 cell viability. (**C**) Effect of MU-PFPS and U-PFPS on H_2_O_2_-induced injury in RAW264.7 cell viability. (**D**) Effects of ROS level on H_2_O_2_-induced injury in RAW264.7 cells pretreated with different concentration of U-PFPS and MU-PFPS. (**E**–**H**) Effect on level of GSH-Px, SOD, CAT activities, and MDA in RAW264.7 cells pretreated with U-PFPS and MU-PFPS; mRNA abundance of *Nrf2* (**I**) and *HO-1* (**J**) were shown. Group without any treatment was remarked as untreated. Concentration of hydrogen peroxide = 544 μM; Concentration of Vc = 100 μg/mL. * *p* < 0.05, ** *p* < 0.01, *** *p* < 0.001; ^#^ *p* < 0.05, ^##^ *p* < 0.01, ^###^ *p* < 0.001 vs. H_2_O_2_ group. Results shown are expressed as means ± SD, * *p* < 0.05, ** *p* < 0.01, *** *p* < 0.001. ns = no significance.

The effect of U-PFPS and MU-PFPS on RAW264.7 cell viability was first assessed using the MTT assay. Results showed that selected concentrations (100–1000 μg/mL) of U-PFPS and MU-PFPS had no toxicity on RAW264.7 cells (Figure 4A). To measure the protective effect against oxidative damage, H_2_O_2_ was used to establish an oxidative damage cell model, which damages cells through direct oxidation or triggers signaling pathways leading to cell death [45]. Different concentrations of H_2_O_2_ were used to treat RAW264.7 cells for 8 h. H_2_O_2_ dose-dependently reduced cell viability, with an IC50 of 544 μM (Figure 4B). Therefore, 544 μM H_2_O_2_ was selected to establish the oxidative damage cell model.

To analyze the protective effects of MU-PFPS and U-PFPS, different concentrations of polysaccharides were used to treat H_2_O_2_-induced oxidative damage in RAW264.7 cells. Compared to U-PFPS treatment groups, MU-PFPS increased cell viability in a dose dependent manner (Figure 4C). MU-PFPS significantly enhanced antioxidant activity at 600 μg/mL, and MU-PFPS with concentrations above 600 μg/mL had stronger protective effects on oxidative damage than U-PFPS. These data partly verified the notion that polysaccharides with higher branching degrees and lower molecular weights possess strong antioxidant activity due to larger surface areas providing greater contact opportunities with radicals or triggering antioxidant reactions [39,46]. Interestingly, both protective effects showed no significant difference at 1000 μg/mL. To determine whether this was due to dose-dependent behavior of U-PFPS or saturation of high-concentration MU-PFPS, dosages of MU-PFPS and U-PFPS were set at 100, 600, and 1000 μg/mL for subsequent oxidative index tests.

ROS lead to oxidative stress, resulting in irreversible damage [47]. When cells are injured by H_2_O_2_, their intracellular ROS oxidize 2′-7′dichlorofluorescin diacetate (DCFH) into dichlorofluorescein (DCF), whose fluorescence intensity is the readout of ROS levels inside the cells [48]. Compared to the H_2_O_2_ model group, pretreatment with U-PFPS and MU-PFPS significantly reduced ROS generation in H_2_O_2_-induced RAW264.7 cells in a dose-dependent manner (Figure 4D). The increase in ROS levels was greatly alleviated at 1000 μg/mL U-PFPS and 600 μg/mL MU-PFPS. Notably, MU-PFPS showed stronger activity in reducing intracellular ROS levels at 600 μg/mL than that of U-PFPS. This suggests that the ability of MU-PFPS to reduce ROS levels was significantly different from that of U-PFPS at concentrations above 600 μg/mL, indicating that protective effects may result from free radical scavenging activity and reducing power. Therefore, we measured antioxidant enzyme activities to identify further variations triggered by structural differences.

### 3.4. U-PFPS and MU-PFPS Attenuates the Oxidative Stress in H_2_O_2_-Induced RAW264.7 Cells

The status of intracellular antioxidant enzymes, including GSH-Px, SOD, CAT, and MDA, serves as an indirect pathway to assess the pro-oxidant/antioxidant balance in tissues as a first line of defense against oxidative stress [32,33]. GSH-Px protects cells from damage caused by peroxides by eliminating superoxide anions [49]. We found that MU-PFPS significantly enhanced enzyme activities in a dose-dependent manner. In contrast, U-PFPS failed to significantly increase GSH-Px activity (Figure 4E). Furthermore, SOD catalyzes the dismutation of superoxide radicals into less harmful molecules to counteract ROS [50,51]. Compared with the U-PFPS group, SOD activities were significantly induced in the MU-PFPS group in a dose-dependent manner (Figure 4F). As CAT scavenges and eliminates hydrogen peroxide [52], both MU-PFPS and U-PFPS significantly increased CAT activity at concentrations above 600 μg/mL (Figure 4G). As an indicator of lipid peroxidation and cell membrane injury, lower MDA levels represent less lipid peroxidation and weaker oxidative stress [53]. MU-PFPS exhibited a dose-dependent effect on decreasing MDA levels, showing significant differences compared to U-PFPS (Figure 4H). These results demonstrate that MU-PFPS protected H_2_O_2_-induced RAW264.7 cells from oxidative damage by comprehensively increasing the activities of GSH-Px, SOD, and CAT, and reducing harmful products such as MDA, thereby aiding cells in recovering from oxidative stress. This enhancement was more evident at higher concentrations. The higher antioxidant activity of MU-PFPS may be related to its lower molecular weight and moderate degree of branching.

Acting as a master sensor of oxidative stress, *Nrf2* binds to antioxidant response elements and activating downstream genes such as *HO-1*, which induces protective responses against oxidative stress [52]. Low concentrations of H_2_O_2_ rapidly upregulate *Nrf2* to compensate for oxidative damage caused by high concentrations of H_2_O_2_. Compared with the untreated group, polysaccharides at 600 μg/mL significantly induced the mRNA expression levels of *Nrf2* and *HO-1* (Figure 4I,J). However, U-PFPS pretreatment at 1000 μg/mL pretreatment significantly decreased the expression levels of *Nrf2* compared to MU-PFPS. The reduced mRNA expression elicited by high concentrations of MU-PFPS and U-PFPS may contribute to similar LPS-induced oxidative stress, but ROS levels were still alleviated by high-concentration polysaccharides. This suggests that the protective effects of MU-PFPS and U-PFPS may be attributed to their free radical scavenging ability and linked to cascade pathways, possibly related to specific structural characteristics. Furthermore, this provides insight into the relationship between ROS scavenging and antioxidant activity. While numerous studies have highlighted the potential applications of polysaccharides in mediating redox reactions, both antioxidants and oxidants can be protective depending on developmental stages and types. Therefore, the relative oxidase and underlying mechanisms of polysaccharides in scavenging reactions may be more critical than scavenging activity alone. Further study is needed to elucidate these specific effects.

## 4. Conclusions

Two different polysaccharides, U-PFPS and MU-PFPS, were extracted from *P. ferulae* using ultrasonic-assisted and microwave/ultrasonic-assisted methods, respectively. The optimized extraction conditions for crude MU-PFPS were determined through single-factor experiments based on polysaccharide content and DPPH radical scavenging ability. Both U-PFPS and MU-PFPS contained glucose, galactose, and mannose, but in different ratios. MU-PFPS had a more balanced composition of glucose, galactose, and mannose, whereas U-PFPS was predominantly composed of glucose. Furthermore, U-PFPS and MU-PFPS display a lack of carboxylic moieties and slight spectral differences appear in the region where C-O-C and C-OH stretching vibrations fall (1200–900 cm^−1^), suggesting different length and branching of chains. Compared with U-PFPS, MU-PFPS exhibited lower molecular weight and a higher tendency to form branches and larger surface areas, which correlated with its stronger antioxidant activity.

In H_2_O_2_-induced oxidative damage in RAW264.7 cells, MU-PFPS demonstrated significantly stronger antioxidant activity in a dose-dependent manner by comprehensively activating antioxidant enzymes compared to U-PFPS. Few differences were observed in antioxidant activity in vitro between U-PFPS and MU-PFPS at low concentrations; this suggests that the dynamic properties of polysaccharides with complex structures may ultimately influence scavenging efficiency and antioxidant-related reactions. Interestingly, both polysaccharides exhibited depressive-like behavior at 1000 μg/mL, linked to ROS-reducing gene expression. This indicates that the antioxidant activity results from structural variations and structure-induced cascade effects on downstream pathways.

Overall, the present study provides a theoretical basis for the understanding of the relationship between structural characteristics and antioxidant activity of polysaccharides. It also supports the hypothesis that different ratios of monosaccharides may affect glycan formation, leading to significant differences in branching degree and molecular weight, which are ultimately reflected in antioxidant activity. Therefore, further investigation into the structure–activity relationship of polysaccharides from *P. ferulae* is warranted.

## Figures and Tables

**Table 1:** Polysaccharide and protein contents of purified polysaccharides.

Content (%)	Ultrasonic-Assisted Polysaccharides				Microwave/Ultrasonic-Assisted Polysaccharides			
	0 mol/L NaCl	0.1 mol/L NaCl	0.2 mol/L NaCl	0.5 mol/L NaCl	0 mol/L NaCl	0.1 mol/L NaCl	0.2 mol/L NaCl	0.5 mol/L NaCl
Yield	3.80 ± 1.06 ^a^	52.37 ± 0.95 ^bc^	33.98 ± 4.45 ^ab^	9.85 ± 4.86 ^b^	5.06 ± 3.47 ^a^	21.53 ± 3.51 ^ab^	54.47 ± 1.74 ^c^	18.94 ± 5.68 ^ab^
Polysaccharide	51.56 ± 2.60 ^bc^	81.83 ± 1.95 ^c^	80.48 ± 1.14 ^c^	47.20 ± 0.47 ^bc^	67.69 ± 3.99 ^bc^	77.45 ± 1.35 ^c^	74.99 ± 0.88 ^c^	61.56 ± 5.06 ^b^
Protein	1.70 ± 3.25 ^a^	17.12 ± 15.65 ^ab^	4.76 ± 5.83 ^a^	8.29 ± 9.86 ^b^	6.53 ± 7.15 ^a^	5.94 ± 6.78 ^a^	4.70 ± 5.09 ^a^	5.23 ± 5.76 ^a^

The same letter of superscript in the same row of data means that the difference is not significant (*p* > 0.05), and different letters mean that the difference is significant (*p* < 0.05).

**Table 2:** Monosaccharides composition and molar ratio of MU-PFPS and U-PFPS.

Polysaccharide	Retention Time (min)			Molar Ratio
	Mannose	Glucose	Galactose	
U-PFPS	21.544	22.086	22.717	3.5:91.1:5.4
MU-PFPS	21.556	21.944	22.612	27.8:39.4:32.8

**Table 3:** Retention time and *M_w_* of MU-PFPS and U-PFPS.

Polysaccharide	Peak No	Retention Time (min)	Mw (kDa)
U-PFPS	1	9.416	1566.68
MU-PFPS	1	10.732	89.26
	2	11.973	14.76

**Table 4:** Retention time and *M_w_* of MU-PFPS separated by Sephadex LH20.

Polysaccharide	Peak No	Retention Time (min)	Mw (kDa)
MU-PFPS	1	10.989	89.9

**Table 5:** Characterization of MU-PFPS and U-PFPS.

Polysaccharide	Z Range	Ra	Standard Deviation	Skewness	Kurtosis
U-PFPS	10.80 nm	0.239 nm	0.612 nm	7.17	80.0
MU-PFPS	5.30 nm	0.200 nm	0.284 nm	2.36	17.6

## Data Availability

The data presented in this study are available on request from the corresponding author.

## Supplementary Materials

The following supporting information can be downloaded at: https://www.mdpi.com/article/10.3390/antiox14010091/s1, Figure S1: Extraction procedure of ultrasonic-assisted polysaccharides from *Pleurotus ferulae*. Figure S2: Extraction procedure of ultrasonic/microwave-assisted polysaccharides from *Pleurotus ferulae.* Figure S3: In vitro antioxidant assays of DPPH· scavenging ability. Two kinds of polysaccharides corresponding to the crude MU-PFPS and U-PFPS. *** *p* < 0.001. Figure S4: MU-PFPS separated by Sephadex LH20 was detected by Alliance HPLC.
