# Supplementary material for: Structure–Antioxidant Activity Relationship of Polysaccharides Isolated by Microwave/Ultrasonic-Assisted Extraction from Pleurotus ferulae
Source: Antioxidants (Basel). 2025 Jan 14;14(1):91. doi: 10.3390/antiox14010091 (PMC11762972; doi:10.3390/antiox14010091)
Supplement: Supplementary file 1 [file antioxidants-14-00091-s001.zip › antioxidants-3352168-supplementary.pdf]

Supplementary Data

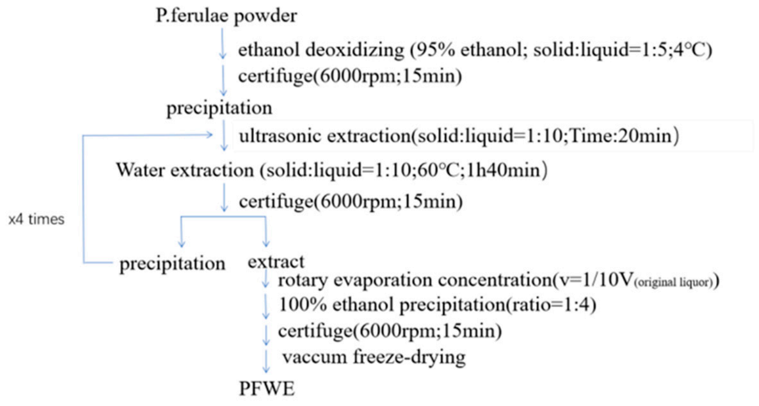

Fig.S1 Extraction procedure of ultrasonic-assisted polysaccharides from *Pleurotus ferulae*

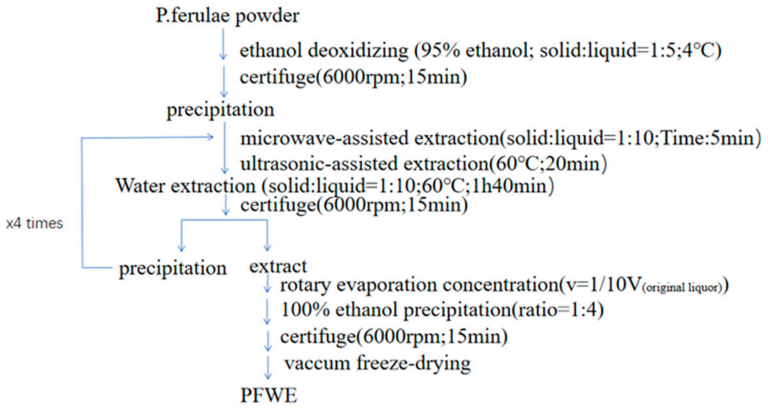

Fig.S2 Extraction procedure of ultrasonic/microwave-assisted polysaccharides from *Pleurotus ferulae*

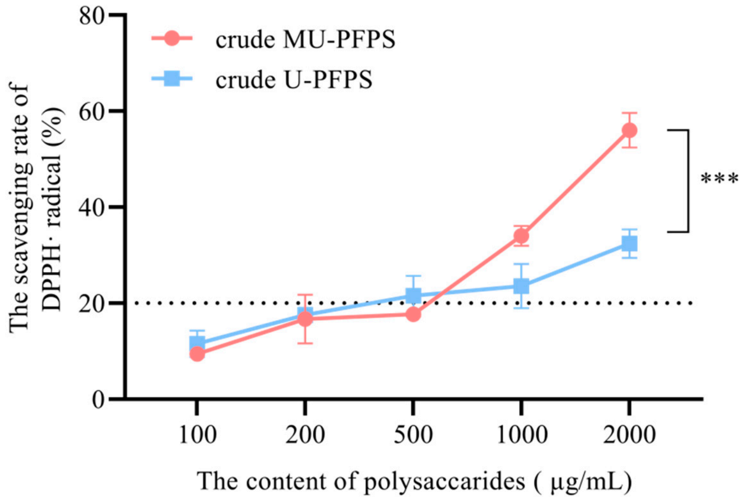

Fig.S3 In vitro antioxidant assays of DPPH· scavenging ability. Two kinds of polysaccharides corresponding to the crude MU-PFPS and U-PFPS. ****p* <*0.001*.

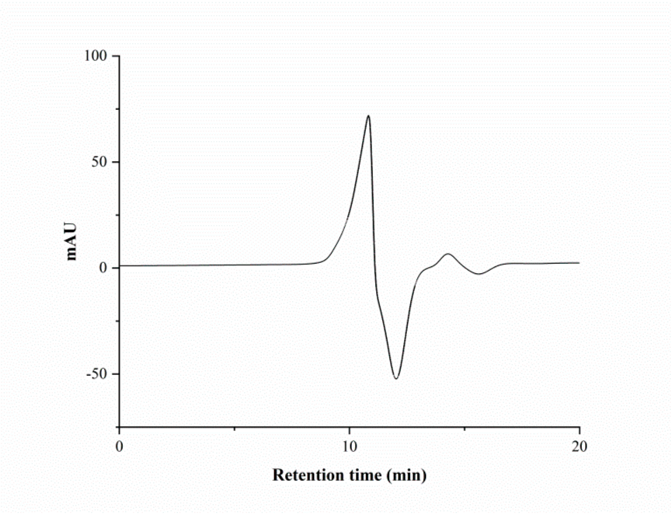

Fig.S4 MU-PFPS separated by Sephadex LH20 was detected by Alliance HPLC.
